# The Scent of Lily Flowers: Advances in the Identification, Biosynthesis, and Regulation of Fragrance Components

**DOI:** 10.3390/ijms26020468

**Published:** 2025-01-08

**Authors:** Yiwei Chen, Xiaoxuan Lu, Ting Gao, Yiwei Zhou

**Affiliations:** 1 College of Forestry and Landscape Architecture, South China Agricultural University, Guangzhou 510642, China; ywchen@scau.edu.cn (Y.C.);; 2 Guangdong Provincial Key Laboratory of Ornamental Plant Germulam Innovation and Utilization, Environmental Horticulture Research Institute, Guangdong Academy of Agricultural Sciences, Guangzhou 510640, China; 3 Xinjiang Field Scientific Observation Research Station of Tianshan Wild Fruit Forest Ecosystem, Yili Botanical Garden, Xinjiang Institute of Ecology and Geography, Chinese Academy of Sciences, Xinyuan 835800, China

**Keywords:** floral scent, lily, *Lilium*, biosynthesis, regulation

## Abstract

Lilies (*Lilium* spp.) are renowned for their diverse and captivating floral scents, which are highly valued both commercially and ornamentally. This review provides a comprehensive overview of recent advancements in the identification, biosynthesis, and regulation of fragrance components in lily flowers. Various volatile organic compounds (VOCs) that contribute to the unique scents of different lily species and cultivars, including terpenoids, benzenoids/phenylpropanoids, and fatty acid derivatives, are discussed. The release patterns of these compounds from different floral tissues and at different developmental stages are examined, highlighting the significant role of tepals. Detection methods such as gas chromatography–mass spectrometry (GC-MS) and sensory analysis are evaluated for their effectiveness in fragrance research. Additionally, the biosynthetic pathways of key fragrance compounds are explored, focusing on the terpenoid and benzenoid/phenylpropanoid pathways and the regulatory mechanisms involving transcription factors and environmental factors. This review also addresses the influence of genetic and environmental factors on fragrance production and proposes future research directions to enhance the aromatic qualities of lilies through selective genetic and breeding approaches. Emphasis is placed on the potential applications of these findings in the floral industry to improve the commercial value and consumer appeal of lily flowers.

## 1. Introduction

Lilies (*Lilium* spp.) encompass all species within the *Lilium* genus of the Liliaceae family. Over 100 wild species of lilies are known; these are primarily distributed in the Northern Hemisphere at latitudes of 10–60°, predominantly in Asia, Europe, and North America [1,2]. Cultivated lilies, hybridized from wild species, are among the world’s most renowned ornamental plants [3]. Currently, over 10,000 lily varieties have been bred worldwide, most deriving from Longiflorum (L), Asiatic (A), Trumpet (T), and Oriental (O) variety groups and their interspecific hybrids (Longiflorum × Asiatic, LA; Oriental × Asiatic, OA; Oriental × Trumpet, OT; Longiflorum × Oriental, LO) [4,5,6]. Lilies possess diverse fragrance types [7] and are highly valued both commercially and ornamentally, meeting significant demand as cut flowers globally, underscoring their importance in the floral industry [8].

Flower fragrance positively impacts human health [9]. Lilies are rich in aromatic compounds and exhibit a wide range of fragrance types. The volatile compounds of different lily species vary significantly [7,10,11]. Sensory fragrance quality is a crucial indicator of lily scent, with different sensory fragrance types possessing distinct major aromatic components [7,12]. The composition, temporal variation, and synthesis regulatory networks of lily floral volatiles have long been popular research topics. Over the past decade, research into lily fragrance has provided us with numerous insights and surprises. Here, we aim to highlight and summarize recent advancements in lily fragrance studies and outline new research directions.

## 2. Release, Detection, and Identification of Lily Floral Compounds

### 2.1. Release of Floral Compounds from Different Tissues and Developmental Stages

Floral scent comprises various volatile organic compounds (VOCs), including terpenoids, benzenoids/phenylpropanoids, fatty acid derivatives, and some amino acid-derived compounds, all of which are detectable in lily flowers [11,13]. Lilies are structured with two whorls of tepals on the outside, two whorls of stamens in the middle, and three fused carpels forming the gynoecium inside [14]. The volatile components and content emitted by the tepals of *Lilium* ‘Siberia’ are similar to those released by the entire flower, while the stamens, pistils, and peduncle tissues release few or no volatile substances. Neutral red staining confirmed that the tepals and their nectaries exhibited the strongest staining [15]. Additionally, methyl benzoate and ethyl benzoate are significant components of the ‘Siberia’ lily fragrance, released by both the outer and inner tepals, but are not detected in the stamens, pistils, leaves, stems, bulbs, or roots, indicating a specific release pattern for these compounds [16]. Studies analyzing the volatile content in the outer and inner tepals, stamens, and carpels of three OT hybrids and two Oriental lily cultivars found significantly higher total volatile molecule content in perianth tissues compared to stamens and carpels, with the outer and inner tepals releasing similar levels of total volatiles [17]. The fragrance composition and release quantity of *Lilium* ‘Siberia’ change along with flower development, peaking during full bloom and decreasing during wilting [18,19]. *Lilium* ‘Novano’ shows a similar pattern of fragrance release [20,21]. Various lily cultivars exhibit similar fragrance release patterns throughout development.

### 2.2. Detection Methods

The sensory evaluation method offers distinct advantages in areas such as consumer preference surveys. However, there are currently very few reports on a sensory analysis of lily fragrance. For instance, Du et al. initially categorized lily fragrance into six types using sensory analysis [7]. Similarly, Aros et al. conducted hedonic scale and floral scent intensity analyses on consumer perceptions of the lily variety ‘Sweetness’ [12]. Recently, we evaluated the fragrance of 36 cut lily varieties using a sensory analysis system and found no significant correlation between aroma intensity and hedonic tone [22]. Owing to its superior qualitative and quantitative capabilities, the primary detection method for lily fragrance remains gas chromatography–mass spectrometry (GC-MS). Most current studies of lily fragrance detection employ headspace solid-phase microextraction (HS-SPME) or adsorbent tube methods to collect the fragrance, followed by analysis using GC-MS (Table 1). However, this method’s requirements for pre-treatment, long detection times, and high costs limit large-scale sample analysis [7,12], hindering efficient fragrance evaluation and the use of genetic studies such as quantitative trait locus (QTL) mapping and genome-wide association studies. As a traditional method, attention has recently shifted to sensory fragrance analysis, which determines fragrance quality and consumer preferences. Moreover, some studies have devised shorter, lower-cost methods for lily fragrance detection. For example, Wei et al. utilized GC-MS and electronic nose (PEN3.5 E-nose, containing 10 metal oxide sensors) analyses to evaluate the postharvest fragrance quality of cut lilies (*Lilium* spp. ‘Manissa’). Their findings revealed that the aroma and flavor intensity peaked on the fourth day after harvest, as marked by significant W2W sensor response values [23]. In a recent study, we also used an E-nose and GC-MS to evaluate the fragrance of 10 cut lily varieties at four blooming stages, finding a good correlation between the E-nose data and GC-MS data, with some sensors being capable of predicting the contents of key VOCs [24]. Moreover, another study demonstrated that the Mach–Zehnder interferometer can monitor lily fragrance in real time by detecting and comparing refractive index changes based on vapor concentrations [25]. These instruments have improved lily fragrance sample detection efficiency to some extent compared to sensory analysis, but the number of studies remains limited, and the research is confined to a few lily varieties. Therefore, focusing on sensory analysis and developing efficient, rapid fragrance detection methods is essential.

Different methods for detecting lily fragrance have their own advantages and disadvantages, with their effectiveness depending on the purpose and specific requirements of the analysis (Table 1). GC-MS can sensitively identify and quantify the specific aroma components of various lily varieties, but the process is time-consuming. Alternatively, the E-nose is a portable device suitable for fast real-time monitoring and distinguishing the floral fragrance components of numerous lily varieties, although it has limited chemical specificity. In practical applications, different methods can be combined to obtain more comprehensive and accurate results.

### 2.3. Identification of Lily Floral Compounds

Lily fragrance consists of numerous volatile compounds, with 1,8-cineole, linalool, (*E*)-*β*-ocimene, and methyl benzoate identified as major components in most lily fragrances [10,15]. The volatile compounds of different lily varieties show significant differences. The two main abundant volatile compounds are monoterpenoids and benzenoids/phenylpropanoids in most fragrant lilies. Commonly fragrant lilies include Oriental lilies, longiflorum lilies, trumpet lilies, and interspecific hybrids like OT and LO lilies. The main aroma components of Oriental lilies are *β*-ocimene, linalool, and methyl benzoate, with some or all of these compounds dominating the fragrance. Secondary volatile compounds include myrcene, (*E*)-*β*-ocimene, *allo*-ocimene, and 1,8-cineole [7,10,11]. *Longiflorum* lilies and LO hybrids share similar scent characteristics, with (*E*)-*β*-ocimene, methyl benzoate, and linalool as the primary aroma components, followed by myrcene, ethyl benzoate, and the fatty acid derivative 2-ethenyl-1,1-dimethyl-3-methylenecyclohexane [7,10]. Trumpet lilies and OT hybrids have nearly identical dominant volatile compounds, with 1,8-cineole being the primary component, followed by methyl benzoate, (*E*)-*β*-ocimene, linalool, and *α*-pinene [10,11,23]. The lightly fragrant LA hybrids’ primary volatiles are monoterpenoids and alkanes [7,10,11]. Asian lilies, which lack fragrance, do not emit any detectable volatiles [10] (Table 2).

Wild lilies, which naturally grow without human cultivation, generally have a fragrance. The main floral volatile of the native Chinese lilies *L. regale* and *L. sulphureum* is 1,8-cineole [1]. The native Japanese lily *L. auratum’*s primary floral volatile is (*E*)-*β*-ocimene [1]. Additionally, *L. sargentiae*’s main volatile is methyl benzoate, whereas *L. bakerianum* var. delavayi primarily emits benzaldehyde, and *L. primulinum* var. ochraceum mainly releases linalool [1] (Table 2).

The floral scent characteristics and components of all lily hybrids reflect their ancestry [1]. For instance, most Asiatic lilies have almost no fragrance, while *L. regale* shares similar scent components with Oriental lilies, suggesting that *L. regale* is a significant ancestor of the Oriental lily fragrance [26,27]. Kong et al. found that the number of overlapping regions of total volatile compounds among different lily hybrids indicates that the LO and OT hybrids combine the scent characteristics of their parents. In contrast, the scent characteristics of LA hybrids, compared to the scentless Asiatic hybrids, may be inherited from *L. longiflorum* [10].

**Table 2:** The mainly volatile compounds of different kinds of *Lilium* species and cultivars.

Type	Group or Species	Cultivar	Floral Scent ^1^	Mainly VOCs ^2^	Class ^3^	Reference
Cultispecies	Oriental Hybrids	‘Viviana’	Scented	*β*-Ocimene, linalool, *allo*-ocimene	Monoterpenoids	[11]
		‘Starfighter’	Scented	Methyl benzoate, linalool, (*E*)-*β*-ocimene	Phenylpropanoids, monoterpenoids	[10]
		‘White Proud’	Scented	Linalool, (*E*)-*β*-ocimene, myrcene	Monoterpenoids	[10]
		‘Pink News’	Scented	*β*-Ocimene, 1,8-cineole, linalool	Monoterpenoids	[11]
		‘Tiber’	Scented	*β*-Ocimene, neo-*allo*-ocimene, methyl benzoate	Phenylpropanoids, monoterpenoids	[11]
	Longiflorum Hybrids	‘Augusta’	Scented	Methyl benzoate, (*E*)-*β*-ocimene, linalool	Phenylpropanoids, monoterpenoids	[10]
		‘White Present’	Scented	(*E*)-*β*-Ocimene, methyl benzoate, myrcene	Phenylpropanoids, monoterpenoids	[10]
		‘White Heaven’	Scented	Methyl benzoate, linalool, ethyl benzoate	Phenylpropanoids, monoterpenoids	[7]
	Longiflorum × Oriental Hybrids	‘Triumphator’	Scented	(*E*)-*β*-Ocimene, methyl benzoate, myrcene	Phenylpropanoids, monoterpenoids	[10]
		‘White Triumph’	Scented	(*E*)-*β*-Ocimene, methyl benzoate, myrcene	Phenylpropanoids, monoterpenoids	[10]
		‘Bell Song’	Scented	Methyl benzoate, linalool, ethyl benzoate	Phenylpropanoids, monoterpenoids	[7]
		‘Pink Brilliant’	Scented	Linalool, methyl benzoate, (*E*)-*β*-ocimene	Phenylpropanoids, monoterpenoids	[7]
		‘Pink Heaven’	Scented	Linalool, methyl benzoate, 2-ethenyl-1,1-dimethyl-3-methylenecyclohexane	Phenylpropanoids, monoterpenoids, fatty acid derivatives	[7]
	Trumpet Hybrids	‘African Queen’	Scented	1,8-Cineole, methyl benzoate, (*E*)-*β*-ocimene	Phenylpropanoids, monoterpenoids	[10]
		‘Pink Planet’	Scented	1,8-Cineole, methyl benzoate, α-pinene	Phenylpropanoids, monoterpenoids	[10]
		‘Regale’	Scented	1,8-Cineole, methyl benzoate, α-pinene	Phenylpropanoids, monoterpenoids	[10]
		‘Yellow Planet’	Scented	1,8-Cineole, (*E*)-*β*-ocimene, methyl benzoate	Phenylpropanoids, monoterpenoids	[10]
	Oriental × Trumpet Hybrids	‘Donato’	Scented	1,8-Cineole, linalool, (*E*)-*β*-ocimene	Monoterpenoids	[10]
		‘Pink Palace’	Scented	1,8-Cineole, (*E*)-*β*-ocimene, methyl benzoate	Phenylpropanoids, monoterpenoids	[10]
		‘Revelation’	Scented	1,8-Cineole, (*E*)-*β*-ocimene, methyl benzoate	Phenylpropanoids, monoterpenoids	[10]
		‘Tabledance’	Scented	1,8-Cineole, (*E*)-*β*-ocimene, linalool	Monoterpenoids	[10]
		‘Palazzo’	Scented	methyl benzoate, *β*-ocimene, 1,8-cineole	Phenylpropanoids, monoterpenoids	[11]
	Longiflorum × Asiatic Hybrids	‘Trebbiano’	Lightly scented	*β*-Ocimene, (*E*)-2-hexanal, methyl benzoate	Phenylpropanoids, monoterpenoids, fatty acid derivatives	[11]
		‘Couplet’	Lightly scented	(*E*)-*β*-Ocimene, DMNT ^4^, methyl benzoate	Phenylpropanoids, monoterpenoids, fatty acid derivatives	[10]
		‘Desiderio’	Lightly scented	(*E*)-*β*-Ocimene, DMNT ^4^, (Z)-ocimene	Monoterpenoids, fatty acid derivatives	[10]
		‘Eyeliner’	Lightly scented	(*E*)-*β*-Ocimene, DMNT ^4^, linalool	Monoterpenoids, fatty acid derivatives	[10]
		‘Serrada’	Lightly scented	(*E*)-*β*-Ocimene, linalool, myrcene	Monoterpenoids	[10]
	Asiatic Hybrids	‘Nello’	Nonscented	-	-	[10]
		‘Navona’	Nonscented	-	-	[10]
		‘Pollyanna’	Nonscented	-	-	[10]
		‘Renoir’	Nonscented	-	-	[10]
Wild species	*Lilium regale*	-	Scented	1,8-Cineole, methyl benzoate, α-pinene	Phenylpropanoids, monoterpenoids	[28]
	*Lilium sulphureum*	-	Scented	1,8-Cineole, methyl benzoate, α-pinene	Phenylpropanoids, monoterpenoids	[1]
	*Lilium auratum*	-	Scented	(*E*)-*β*-Ocimene, methyl benzoate, linalool	Phenylpropanoids, monoterpenoids	[27]
	*Lilium sargentiae*	-	Scented	Methyl benzoate, 1,8-cineole, (*E*)-*β*-ocimene	Phenylpropanoids, monoterpenoids	[26]
	*Lilium bakerianum* var. *delavayi*	-	Scented	Benzaldehyde, linalool, (*E*)-*β*-ocimene	Benzenoids, monoterpenoids	[26]
	*Lilium primulinum* var. *ochraceum*	-	Scented	Linalool, 1,8-cineole, *β*-elemene	Monoterpenoids, sesquiterpenoids	[26]

^1^ The floral scent intensity was evaluated in previous articles that have been published. ^2^ The top three most abundant compounds in terms of total volatiles. ^3^ The classification of mainly volatile compounds. ^4^ (*E*)-4,8–dimethyl–1,3,7-nonatriene.

### 2.4. Key VOCs Influencing Lily Fragrance

In studies on the sensory concentration of lilies, although the release amounts of lily fragrance compounds are positively correlated with fragrance intensity [26] and the release patterns of terpenoids in different lily varieties are consistent with fragrance concentration, a more intense fragrance does not necessarily make lilies more popular [12]. This indicates that sensory fragrance type is an important indicator of lily fragrance quality. Regarding the study of the sensory fragrance types of lilies, Du et al. [7] preliminarily classified lily fragrances into types such as weak fragrance, cool fragrance, fruity fragrance, musky fragrance, honey fragrance, and lily fragrance. The contents of compounds such as 1,8-cineole, linalool, (*E*)-*β*-ocimene, myrcene, and methyl benzoate vary significantly among different fragrance types. Although weakly fragrant lilies also contain important fragrance components like linalool and methyl benzoate, they are almost undetectable by the human nose. Cool-scented lilies have higher levels of cineole. Our recent study showed no significant correlation between the sensory concentration of lilies and hedonic tone. However, the percentage of linalool is significantly positively correlated with the hedonic tone of lily fragrance, while the percentage of methyl salicylate is significantly negatively correlated with the hedonic tone [22]. Nevertheless, the specific compounds and sensory characteristics that control the formation of different sensory fragrance types remain unclear.

## 3. Biosynthesis of Lily Fragrance Components

To date, research on the synthesis pathways of lily fragrance volatiles has mainly focused on terpenoids, benzenoids/phenylpropanoids, and fatty acid derivatives, with significant progress in the biosynthesis of terpenoids in recent years (Table 3).

### 3.1. Biosynthesis Pathways of Terpenoids

Terpenoid volatiles are the most abundant components of lily fragrance, mainly including monoterpenoids and sesquiterpenoids [7,29]. These are formed via two pathways: the C5 precursor of monoterpenoids is synthesized through the methylerythritol phosphate (MEP) pathway in plastids, derived from pyruvate and glyceraldehyde-3-phosphate (G3P). One molecule of dimethylallyl diphosphate (DMAPP) and one molecule of IPP condense head-to-tail to form geranyl diphosphate (GPP), which is then catalyzed by monoterpenoid synthase to produce monoterpenoids [7], such as (*E*)-*β*-ocimene and myrcene. The cytosolic mevalonate (MVA) pathway, derived from acetyl-CoA, synthesizes isopentenyl diphosphate (IPP), and two molecules of IPP and one molecule of DMAPP condense to form farnesyl diphosphate (FPP), which is then catalyzed by sesquiterpenoid synthase to produce sesquiterpenoids [30].

High activation levels of the genes encoding *DXS*, *DXR*, *HDS*, *HDR*, *IDI*, and *GPS*/*GPPS* in the upstream MEP pathway promote the synthesis of monoterpenoid volatiles [20]. Transferring *LiDXS* and *LiDXR* resulted in increased levels of monoterpenoids and sesquiterpenoids such as caryophyllene and linalool in tobacco [31]. *LiMCT* from *Lilium* ‘Sorbonne’ plays a crucial role in regulating the synthesis of monoterpenoids and other isoprenoid precursors in transgenic *Arabidopsis* [32]. Additionally, Cao et al. identified the genes related to terpenoid biosynthesis, such as *LiDXS2*, *LiLIS*, and *LiMYS*, in *Lilium* ‘Sorbonne’, and confirmed their involvement in monoterpenoid biosynthesis through heterologous transformation in *Arabidopsis* [33]. In the transcriptome of *Lilium* ‘Siberia’, the *MCT*, *CMK*, and *MDS* genes encoding enzymes in the MEP pathway were identified, along with six genes encoding enzymes in the MVA pathway, including two *AACT*, one *HMGS*, two *HMGR*, and one *MK*. The expression of these key genes varies with floral scent changes but is more complex during the circadian rhythm [21]. In studies on the gene function of terpenoid synthases, the expression levels of *TPS* genes in seven lily varieties were strongly positively correlated with the content of monoterpenoids such as linalool and *β*-ocimene [11]. In the transcriptome of *Lilium* ‘Siberia’, 19 *TPS* genes were identified, including linalool synthase (*LIS*), (*E*)-*β*-ocimene synthase, myrcene synthase, and sesquiterpenoid (3S,6E)-nerolidol synthase (*NES*) genes. Most of these *TPS* gene expression changes are related to terpenoid biosynthesis during flower development and the circadian rhythm [21]. In enzyme function studies, most researchers have chosen the fragrant ‘Siberia’ lily as a model plant to study the molecular mechanisms of lily fragrance biosynthesis. Five *TPS* genes have been successfully cloned, including *LoTPS1*, which is localized in plastids and catalyzes the formation of (*Z*)-*β*-ocimene and linalool from GPP, and *LoTPS3*, which is localized in mitochondria and catalyzes the formation of linalool from GPP and *cis*-nerolidol from FPP [19]. *LoTPS2*, localized in the cytoplasm, catalyzes the formation of (*E*,*E*)-*α*-farnesene from FPP, while *LoTPS4*, a multi-product enzyme localized in plastids, catalyzes the formation of D-limonene and *β*-ocimene from GPP and *trans*-bergamotene from FPP [18]. *LoTPS5*, localized in plastids, catalyzes the formation of squalene from FPP [34]. Other teams have also successfully isolated *LiTPS2* from ‘Siberia’, a terpene synthase localized in the chloroplasts that catalyzes the formation of linalool and *trans*-nerolidol from GPP and FPP, respectively. Overexpressing ‘Siberia’ *LiTPS2* in tobacco significantly increased the release of linalool, myrcene, and (*E*)-*β*-ocimene [35]. Additionally, ‘Sorbonne’ (LsoTPS-1, LsoTPS-2, and LsoTPS-3) and ‘Red Life’ (LrlTPS-1 and LrlTPS-3) were able to convert GPP into (*E*)-*β*-ocimene, α-pinene, and limonene.

### 3.2. Biosynthetic Pathways of Benzenoid/Phenylpropanoids

Benzenoid/phenylpropanoid volatiles are the second-largest category of floral volatile organic compounds in plants [13]. The shikimate pathway converts the aromatic amino acid phenylalanine (Phe) into cinnamic acid (CA) via phenylalanine ammonia-lyase (PAL) [36,37,38]. In lily fragrance, cinnamic acid can be converted into the precursor benzoic acid through the *β*-oxidation and non-*β*-oxidation pathways [39,40], which is then catalyzed by enzymes such as BSMT and BAHD to produce methyl benzoate and ethyl benzoate [16]. In other plant volatile synthesis pathways, CA can also be hydroxylated by cinnamate-4-hydroxylase (C4H) to form p-coumarate, which is further modified to produce a series of volatile benzenoids/phenylpropanoids [41,42,43].

PAL catalyzes the deamination of phenylalanine to form CA, the first step in phenylpropanoid biosynthesis [36,37]. The transcriptome of *Lilium* ‘Siberia’ contains seven *PAL* genes, the expression of which is consistent with the release of benzenoid/phenylpropanoid compounds during flower development but remains almost unchanged during the circadian rhythm [21]. CA is further modified through the *β*-oxidation and non-*β*-oxidation pathways to form benzenoids/phenylpropanoids [13]. In the *β*-oxidation pathway of petunias, four reactions are catalyzed by CNL/AAE (cinnamate-CoA ligase/acyl-activating enzyme), CHD (cinnamoyl-CoA hydratase-dehydrogenase), and KAT (3-ketoacyl-CoA thiolase) [44,45,46]. Two *KAT* genes were found in *Lilium* ‘Siberia’, and their expression slightly increased after flower development [21]. Key genes in the BSMT family play a direct role in the production and release of methyl benzoate in lily fragrance [47]. Researchers have found that the BAHD family member *LoAAT1* in *Lilium* ‘Siberia’ is not only the preferred candidate gene for ethyl benzoate production but is also the first to be reported as participating in the biosynthesis of methyl benzoate [16].

**Table 3:** Lily flower fragrance-related synthase genes, validated through in vitro enzyme-catalyzed reactions or transgenic verification.

Variety	Aroma Intensity	Gene	Method	Precursor/Transformed Plant	Major Products/Impact	References
‘Siberia’	Strong	*LoAAT1*	In vitro enzyme-catalyzed reaction	Ethanol and benzoyl-CoA/methanol and benzoyl-CoA	Ethyl benzoate/methyl benzoate	[16]
‘Siberia’	Strong	*LoTPS1*	In vitro enzyme-catalyzed reaction	GPP	(*Z*)-*β*-Ocimene and linalool	[19]
‘Siberia’	Strong	*LoTPS3*	In vitro enzyme-catalyzed reaction	GPP/FPP	Linalool/*cis*-nerolidol	[19]
‘Siberia’	Strong	*LoTPS2*	In vitro enzyme-catalyzed reaction	FPP	(*E*,*E*)-*α*-farnesene	[18]
‘Siberia’	Strong	*LoTPS4*	In vitro enzyme-catalyzed reaction	GPP/FPP	D-limonene and *β*-myrcene/*(E)*-*α*-bergamotene	[18]
‘Siberia’	Strong	*LoTPS5*	In vitro enzyme-catalyzed reaction	FPP	Squalene	[34]
‘Siberia’	Strong	*LiTPS2*	In vitro enzyme-catalyzed reaction	GPP/FPP	Linalool/*trans-*nerolidol	[35]
‘Red life’	Light	*LrlTPS-1*	In vitro enzyme-catalyzed reaction	GPP	(E)-β-Ocimene, *α*-pinene, and limonene	[7]
‘Red life’	Light	*LrlTPS-3*	In vitro enzyme-catalyzed reaction	GPP	(E)-β-Ocimene, *α*-pinene, and limonene	[7]
‘Sorbonne’	Strong	*LsoTPS-1*	In vitro enzyme-catalyzed reaction	GPP	(E)-β-Ocimene, *α*-pinene, and limonene	[7]
‘Sorbonne’	Strong	*LsoTPS-2*	In vitro enzyme-catalyzed reaction	GPP	(E)-β-Ocimene, *α*-pinene, and limonene	[7]
‘Sorbonne’	Strong	*LsoTPS-3*	In vitro enzyme-catalyzed reaction	GPP	(E)-β-Ocimene, *α*-pinene, and limonene	[7]
‘Siberia’	Strong	*LiDXS*	Heterologous transformation	Tobacco	Increased levels of sclareol and caryophyllene	[31]
‘Siberia’	Strong	*LiDXR*	Heterologous transformation	Tobacco	Increased levels of sclareol, linalool, and caryophyllene	[31]
‘Sorbonne’	Strong	*LiMCT*	Heterologous transformation	*Arabidopsis*	Increased levels of *AtTPS14* expression; increased carotenoid and chlorophyll content	[32]
‘Sorbonne’	Strong	*LiLIS, LiMYS*	Heterologous transformation	*Arabidopsis*	Significant changes in the gene expression of MVA and MEP pathways	[33]

### 3.3. Biosynthetic Pathways of Fatty Acid Derivatives

In addition, a small quantity of volatile fatty acid derivatives was detected in the floral scent of lilies. The precursors of these derivatives are fatty acids produced from phospholipids, triacylglycerols, and glycolipids by acyl hydrolases, which are then metabolized via the lipoxygenase (LOX) pathway [13,48]. Subsequently, these fatty acids undergo a series of reactions to form esters, amides, and acyl chlorides, which are volatile fatty acid derivatives [42,49]. Although these volatile compounds are present in minor amounts, they still significantly influence the floral aroma of lilies.

In the upstream biosynthetic pathways of these volatile fatty acid derivatives, 4 *LOX* and 14 alcohol dehydrogenase (*ADH*) genes were identified. *LOX* gene expression peaks during the full-bloom stage and declines as the flower ages. The 14 *ADH* genes can convert C6 and C9 aldehydes into volatile alcohols [49]. Furthermore, researchers used WGCNA to analyze the correlation between the differentially expressed genes (DEGs) related to floral scent variations during the development and diurnal rhythm of *Lilium* ‘Siberia’ and individual flower volatiles, identifying 90 potential key genes involved in the biosynthesis of fatty acid derivatives [21].

## 4. Regulatory Mechanisms of Lily Floral Scent Components

The release of floral volatiles is regulated not only by the plant’s internal physiological mechanisms but also by various environmental factors [50], which can influence the emission rate and composition of volatiles [51].

### 4.1. Transcriptional Regulation of Floral Scent Component Biosynthesis

The transcriptional regulation of floral scent component biosynthesis involves multiple molecular mechanisms and regulatory factors. However, in studies on the transcriptional regulation of lily floral scent, only a limited number of transcription factors have been identified as having transcriptional regulatory effects on some key floral scent synthase genes (Table 4). In the floral volatiles of *Lilium* ‘Siberia’, 1270 transcription factors (TFs) from 55 families, including bHLH, ERF, NAC, MYB, C2H2, MYB, and bZIP, have been predicted [21]. Thirty-one TFs have been identified as potentially positively regulating the production of monoterpenoid volatiles in *Lilium* ‘Sorbonne’, with many belonging to the bHLH, bZIP, and MYB families [52]. LibHLH22 and LibHLH63 significantly promote the expression of *LiDXR* and *LiTPS2*, thereby enhancing floral scent release [53]. LiNAC100 has a novel function in regulating floral scent by directly controlling the linalool synthase gene *LiLIS* [54]. LiMYB1 forms transcriptional regulatory complexes with LiMYB308 and LiMYB330, jointly enhancing the activation of the *LiTPS2* promoter, thereby regulating the synthesis of terpenoid floral scent compounds in *Lilium* ‘Siberia’ [55]. LiMYB305 directly binds to and activates the *LiOCS* promoter, increasing monoterpene synthesis [56]. The MYB transcription factor LiSRM1 from ‘Siberia’ petals negatively regulates the synthesis of major monoterpenoid volatiles [57]. The R2R3-MYB transcription factor *LhODO1* is expressed only in the perianth, the main source of floral scent, with a circadian rhythm consistent with the expression rhythms of the shikimate pathway and *PAL* genes, suggesting a key role in regulating the production of volatile benzenoids/phenylpropanoids in lilies [58].

### 4.2. Circadian Rhythm

The biosynthesis of lily floral volatiles is controlled by the plant’s circadian rhythm. Studies have shown that the emission of volatile compounds is significantly higher at night than during the day in eight cultivars from different hybrid combinations. Even scentless varieties like ‘Tresor’ and ‘Ceb Dazzle’ emit small amounts of volatiles at night, despite almost no emission during the day [15]. The total emission of volatiles from native Chinese wild lilies, such as *L. regale*, *L. ludingensis*, and *L. concolor*, is richer at night, being approximately 5 to 40 times higher than during the day [10]. Many monoterpenoid compounds are attractants for nocturnal moths, and the nocturnal bias in lily floral scent release is thought to enhance pollination success by attracting night-time visitors [26].

### 4.3. Light

Light is an important environmental signal for plant growth, development, and secondary metabolite production. Light quality affects volatile emissions [59]. For instance, snapdragons release large amounts of terpenoid volatiles under blue light [60]. However, in lilies, studies have mainly focused on how light quality affects the growth, photosynthetic characteristics, and endogenous hormones of tissue-cultured seedlings [61]. Research on light intensity has shown that the quantity and release of lily floral scent components first increase and then decrease under light intensities of 0, 100, 300, 600, 1000, and 1500 μmol m^−2^ s^−1^ [62]. After transitioning from darkness to light, the emission of major floral scent components such as linalool and ocimene increases, while transitioning from light to darkness decreases the volatile content. Further studies have isolated a nuclear-localized light signal factor, LoCOP1, which negatively regulates the production and release of lily floral volatiles [50].

### 4.4. Hormones

Many plant VOCs are precursors, chemical analogs, or structural homologs of typical plant growth regulators such as auxins, cytokinins (CKs), gibberellins (GAs), and abscisic acid (ABA) [63]. Plant hormones can enhance or induce the emission of plant volatiles [63]. Treating ‘Siberia’ lily petals with 200 and 600 μmol/L methyl jasmonate (MeJA) solutions significantly increased the release of floral volatiles [64]. Similarly, treatment with 300 mg/L ABA significantly increased the total release of floral volatiles in *Lilium* ‘Sorbonne’ [65].

### 4.5. Other Factors

In addition to light, circadian rhythm, and hormones, many other factors can influence the release of lily floral scent. For example, 2-(aminooxy) acetic acid (AOA), an inhibitor of phenylalanine ammonia-lyase, significantly reduces volatile emissions when sprayed continuously during the bud stage, without affecting flower opening [66]. The concentration of floral volatiles in lilies is altered by the nitrogen and potassium nutrients provided during cultivation. Nitrogen fertilization increases the concentration of terpenoids such as linalool, while low potassium levels increase the concentration of methyl benzoate [66]. Researchers have also found more refined regulatory mechanisms in lily floral scent formation. Increased light intensity triggers Ca^2+^ influx into the cytoplasm, activating the downstream gene expression of monoterpene synthases, thereby regulating the biosynthesis and release of monoterpenoids [67]. Hexokinase (LoHXK) and fructokinase (LoFRK) in lilies significantly alter the release of *β*-ocimene and linalool by regulating the expression of the volatile synthesis genes *LoTPS1* and *LoTPS3* [68]. Additionally, temperature is a crucial factor affecting the synthesis and release of volatiles [69]. At 30 °C, the release of terpenoids, alcohols, aldehydes, ketones, and esters in *Lilium* ‘Siberia’ reaches its maximum, while the release of alkanes is minimal [62].

## 5. Conclusions and Prospects

**Figure 1:**
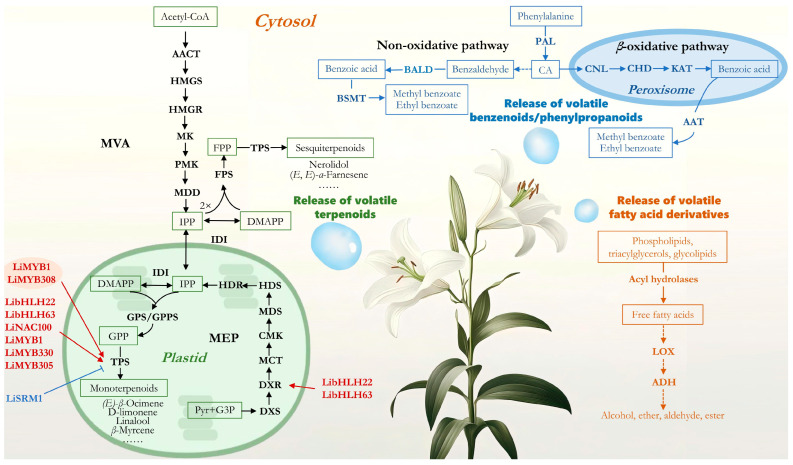
Possible biosynthetic pathways and verified transcriptional regulators of lily fragrance. The red TFs indicate positive regulation, while the blue TFs indicate negative regulation. The orange-red ellipse encompassing LiMYB1 and LiMYB308 indicates that they only exert transcriptional regulatory effects on *TPS* when they form a complex. MEP is the pyruvate and glyceraldehyde-3-phosphate (G3P)–derived 2-C-methyl-D-erythritol-4-phosphate pathway; DXS, 1-deoxy-D-xylulose 5-phosphate synthase; DXR, 1-deoxy-D-xylulose 5-phosphate reductase; MCT, MEP cytidyltransferase; CMK, 4-diphosphocytidyl-2-C-methyl-D-erythritol kinase; MDS, 2-C-methyl-D-erythritol 2,4-cyclodiphosphate synthase; HDS, (*E*)-4-hydroxy-3-methyl-but-2-enyl diphosphate synthase; HDR, (*E*)-4-hydroxy-3-methyl-but-2-enyl diphosphate reductase; IPP, isopentenyl diphosphate; IDI, isopentenyl diphosphate isomerase; DMAPP, dimethylallyl diphosphate; GPS/GPPS, GPP synthase; GPP, geranyl diphosphate; TPS, terpene synthase; MVA, the cytosolic mevalonate pathway originating from acetyl-CoA; AACT, acetoacetyl-CoA thiolase; HMGS, 3-hydroxy-3-methylglutaryl-CoA synthase; HMGR, 3-hydroxy-3-methylglutaryl-CoA reductase; MK, mevalonate kinase; PMK, phosphomevalonate kinase; MDD, mevalonate-5-diphosphate decarboxylase; FPS, FPP synthase; FPP, farnesyl diphosphate; PAL, phenylalanine ammonialyase; CA, cinnamic acid; BALD, benzaldehyde dehydrogenase; BSMT, benzoicacid/salicylicacidcarboxylmethyltransferase; CNL, cinnamoyl-CoA ligase; CHD, cinnamoyl-CoA hydratase-dehydrogenase; KAT, 3-ketoacyl CoA thiolase; AAT, alcohol acyltransferase; LOX, lipoxygenase; ADH, alcohol dehydrogenase.

Floral scent is a crucial factor in encouraging consumers to purchase lilies. Therefore, breeders are dedicated to developing new lily varieties with more intense and long-lasting fragrances through genetic improvement and breeding techniques. For instance, crossing scentless Asiatic hybrid lilies with *L. longiflorum* has successfully produced varieties with pleasant aromas [3]. However, controlling the fragrance traits of offspring precisely during hybrid breeding is challenging, due to the complex synthesis pathways and regulatory mechanisms of floral scent components. Current research mainly focuses on the terpenoid synthesis pathway, with a limited understanding of the gene functions and molecular regulatory mechanisms of other floral scent components’ metabolic pathways [13,70]. In recent years, significant progress has been made in studying the release and synthesis regulation of important compounds in lily fragrance. Lily fragrance comprises many volatile compounds, with 1,8-cineole, linalool, (*E*)-*β*-ocimene, and methyl benzoate being the primary compounds in most lily varieties [10]. Fatty acid derivatives like geranyl acetate and amino acid derivatives have also been identified [7]. Functional gene studies have elucidated several key genes and transcription factors involved in volatile synthesis pathways [11,16,53,54]. A schematic diagram illustrating the biosynthesis of lily fragrance components and their regulation by transcription factors is shown in Figure 1. However, these studies primarily focus on a limited number of varieties (‘Siberia’ and ‘Sorbonne’), and the prolonged absence of reference genome sequences has hindered progress in deciphering the gene regulatory network of lily fragrance and in the genetic engineering of breeding. The recent publication of the large lily genome sequence [71] marks a milestone that will significantly accelerate lily fragrance research, providing an opportunity to identify the key genes involved in the formation of different lily fragrances.

Moreover, the lack of efficient and stable genetic transformation systems applicable to various lily varieties is another major limitation in the discovery and application of lily fragrance genes. Some successful cases have been reported, such as a novel *Agrobacterium transformation* method, based on injection in the meristems, using *L. regale* [72], the optimized genetic transformation systems for *Lilium* ‘Siberia’ and *Lilium* ‘Sorbonne’ [73], and the genetic transformation system for *L. pumilum* constructed by Zhang et al., which preliminarily verified the function of LpNAC6 in abiotic stress [74]. The successful construction of the CRISPR/Cas9 system for lilies [75] also provides potential tools for functional gene discovery and new germplasm creation. However, the effectiveness of these tools and systems varies significantly among the different genotypes [76], and obtaining positive gene-edited seedlings through CRISPR/Cas9 remains rare, making the construction and optimization of genetic transformation and gene-editing systems for different lily varieties still challenging.

In the field of floral scent detection, several highly efficient techniques, such as gas chromatography–ion mobility spectrometry (GC-IMS) [77], comprehensive two-dimensional gas chromatography–time-of-flight mass spectrometry (GC × GC-TOF-MS) [78], fourier transform ion cyclotron resonance mass spectrometry (FT-ICR-MS) [79], proton transfer reaction time-of-flight mass spectrometry (PTR-TOF-MS) [80], and the GC-PTR-TOF-MS method [81], can be further developed and applied to the detection of lily fragrance. These techniques allow for the comprehensive identification of the various VOCs of lily fragrance. Moreover, special instruments like the Mach–Zehnder interferometer can detect spectral changes and could be optimized in the future to develop methods for visualizing fragrance detection [25].

Future research on lily fragrance can be conducted in several directions, based on the newly published genome sequence:

(1) Identify the key aroma compounds determining sensory fragrance types, using sensory omics techniques.
(2) Develop efficient and rapid detection systems for lily fragrance.
(3) Combine multi-omics and molecular biology to deeply analyze the synthesis and regulatory mechanisms of floral scent substances.
(4) Construct genetic maps to achieve gene localization and the positional cloning of fragrance traits.
(5) Develop the molecular markers significantly associated with lily fragrance.
(6) Build genetic transformation and gene-editing systems that are suitable for different lily varieties.
(7) Explore the impact of environmental factors on lily fragrance.
(8) Enhance research on the application of lily fragrance in the daily chemical industry.
(9) Construct a pan-genome for lilies.

These studies will help precisely identify lily materials with specific aroma substances, create new germplasm and varieties with pleasant fragrances, promote the development and upgrading of the lily industry, and bring more attractive fragrances and health to people’s lives.

## Figures and Tables

**Table 1:** Summary of lily fragrance detection methods.

Fragrance Collection Method	Fragrance Analysis Method	Advantage	Disadvantage	References
Adsorbent tube	GC-MS	Qualitative and quantitative analysis of VOCs	Multiple steps, relatively long analysis time	[1,3,10,15,20]
SPME	GC-MS			[7,11,12,16,18,19,21,25,26]
-	Sensory	Directly reflects consumer perception of fragrance	High labor costs, strong subjectivity	[7,12,22]
-	E-nose	Real-time detection, short analysis time	Cannot qualitatively analyze VOCs	[23,24]
-	Metal-oxide semiconductor odor sensor	Real-time detection, short analysis time		[26]
-	Mach–Zehnder interferometer	Real-time visualization of scent accumulation		[25]

**Table 4:** The transcription factors and target genes related to ‘Siberia’ lily fragrance that have been validated through experiments.

Transcription Factor	Target Gene	Regulatory Mode	Controlled VOCs	References
LibHLH22	*LiDXR*, *LiTPS2*	Positive	Linalool, (*E*)-*β*-ocimene	[53]
LibHLH63	*LiDXR*, *LiTPS2*	Positive	Linalool, (*E*)-*β*-ocimene	[53]
LiNAC100	*LiLiS*	Positive	Linalool	[54]
LiMYB1	*LiTPS2*	Positive	Linalool, (*E*)-*β*-ocimene	[55]
LiMYB305	*LiTPS2*	Positive	Linalool, (*E*)-*β*-ocimene	[55]
LiMYB330	*LiTPS2*	Positive	Linalool, (*E*)-*β*-ocimene	[55]
Complex of LiMYB1 and LiMYB308	*LiTPS2*	Positive	Linalool, (*E*)-*β*-ocimene	[55]
LiMYB305	*LiOcS*	Positive	(*E*)-*β*-Ocimene	[56]
LiSRM1	*LiLiS*, *LiOcS*	Negative	Linalool, (*E*)-*β*-ocimene	[57]
